# Data on the optimization of behavioral tasks for senescence-accelerated mouse prone 8 (SAMP8)

**DOI:** 10.1016/j.dib.2016.05.044

**Published:** 2016-05-26

**Authors:** Shuichi Yanai, Shogo Endo

**Affiliations:** Aging Neuroscience Research Team, Tokyo Metropolitan Institute of Gerontology, Japan

**Keywords:** SAMR1, SAMP8, Memory, Fear conditioning, Object recognition

## Abstract

This data article contains the supporting information for the research article entitled “Early onset of behavioral alterations in senescence-accelerated mouse prone 8 (SAMP8)” [1]. Senescence-accelerated mouse prone 8 (SAMP8), which originally developed from AKR/J mice, shows learning and memory impairments at the age of 8–12 months. However, little information is still available on phenotypical characteristics of younger SAMP8. To fully understand the phenotype of younger SAMP8, we optimized two behavioral tasks for SAMP8. In the object recognition task, 4-month-old SAMP8 made significantly more contacts with the familiar objects compared to age-matched SAMR1, however, distance traveled for both strains of mice were comparable. In the fear conditioning task, conventionally-used CS–US combination failed to induce robust conditioned fear in both strains of mice.

**Specifications Table**

**Table:** 

Subject area	*Biology*
More specific subject area	*Behavioral neuroscience*
Type of data	*Figure, table*
How data was acquired	*Behavioral phenotyping*
Data format	*Raw and analyzed*
Experimental factors	*Four-month-old SAMP8 and age-matched control SAMR1 mice*
Experimental features	*Phenotypical characteristics were examined using the object recognition task and the Pavlovian fear conditioning task*
Data source location	*Aging Neuroscience Research Team, Tokyo Metropolitan Institute of Gerontology, Itabashi, Tokyo*
Data accessibility	*Data are supplied with this article*

Value of the data

• Four objects were selected to be used in the object recognition task. These objects had no significant innate preference and could be used broadly in mouse behavioral studies.
• In the object recognition task, the benchmark data for SAMR1 and SAMP8 was obtained for the future comparison.
• The conventional conditioning protocol in the fear conditioning task failed to induce conditioned fear in 4-month-old SAMP8 and SAMR1. This is valuable to avoid the conventional protocol to induce fear memory in these mice.

## 1 Data

**Fig. 1:**
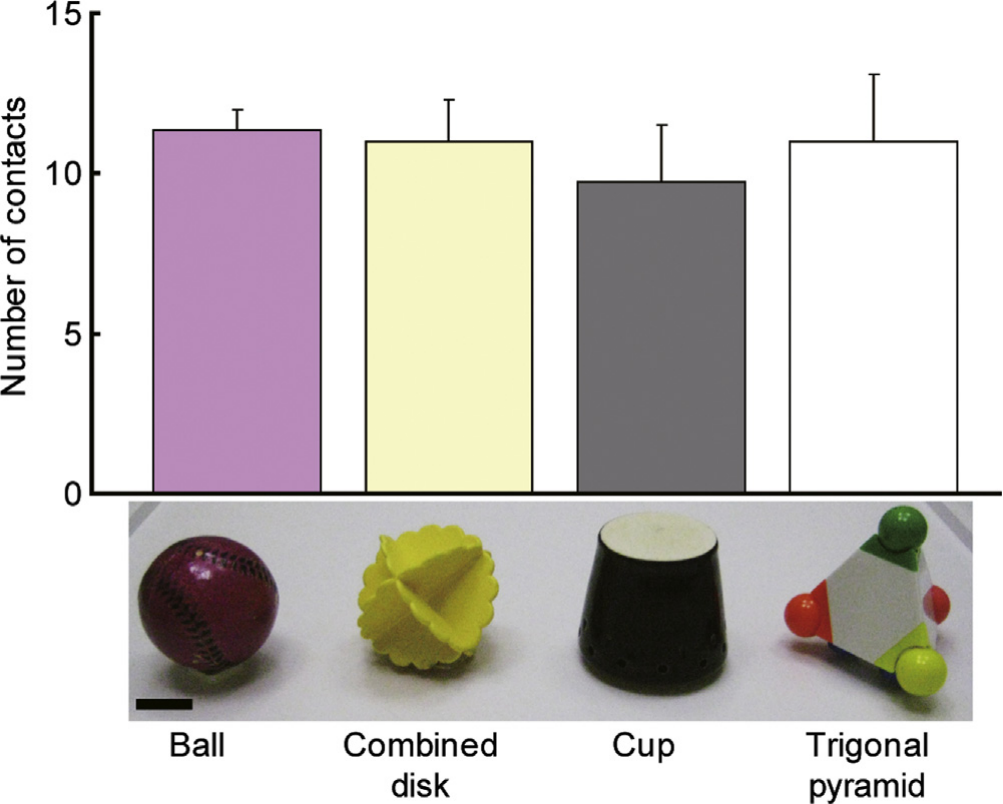
Object preference pilot test. A mouse was allowed to explore the arena containing the ball, the combined disk, the cup, and the trigonal pyramid for 10 min to examine if these 4 objects were equally preferred by the mice. One-way ANOVA confirmed that there was no significant preference for a particular object among the 4 objects (Fig. 1; *F* (3, 31)=0.21, n.s.). These objects had similar volumes, but were completely different in luminosity, shape, and surface texture. Scale bar indicate 2 cm. Error bars indicate S.E.M.

**Fig. 2:**
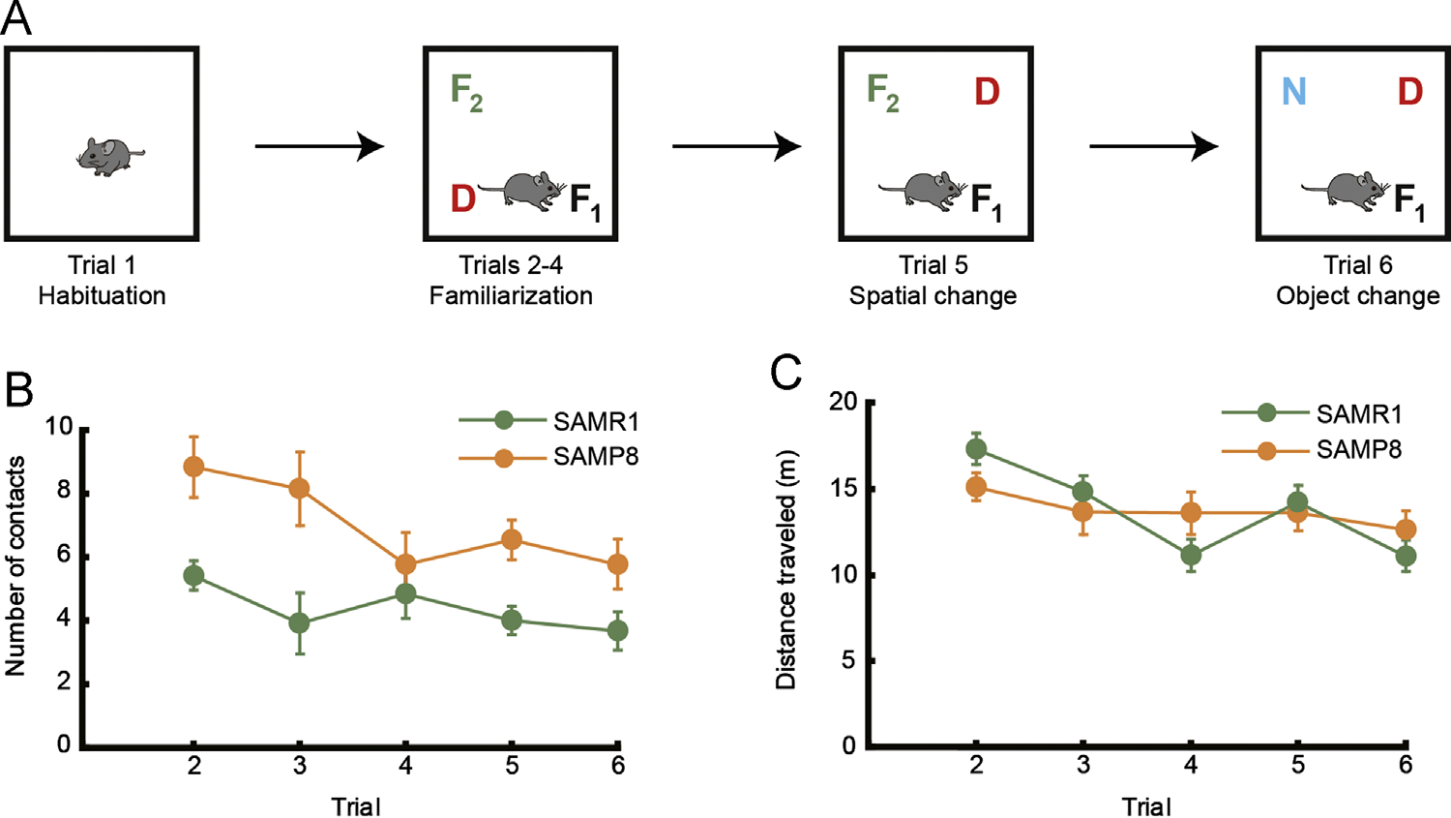
Object recognition task. (A) Schematic diagram of the object recognition task. After habituating to the empty arena (trial 1), 4-month-old SAMR1 (*n*=11) and SAMP8 (*n*=13) were allowed to explore the object for five successive 5-min trials (trials 2–6). (B) Number of contacts with the familiar object (*F*_1_), which remained in the same location throughout the entire experiment. A mixed design two-way ANOVA (with strain as the between-subject factor and training trial as the within-subject factor) revealed a significant main effect of trial (*F* (4, 88)=5.04, *p*<0.001; Table 1), indicating that there was a significant decrease in the number of contacts made with the familiar object for both strains over time. However, overall SAMP8 made significantly more contacts with the objects than SAMR1 (*F* (1, 22)=13.08, *p*<0.01; Table 1). (C) The distance traveled during the entire experiment. A two-way ANOVA revealed that both strains of mice traveled comparable distances throughout the experiment (*F* (1, 22)=0.00, n.s.; Table 1). Error bars indicate S.E.M.

**Fig. 3:**
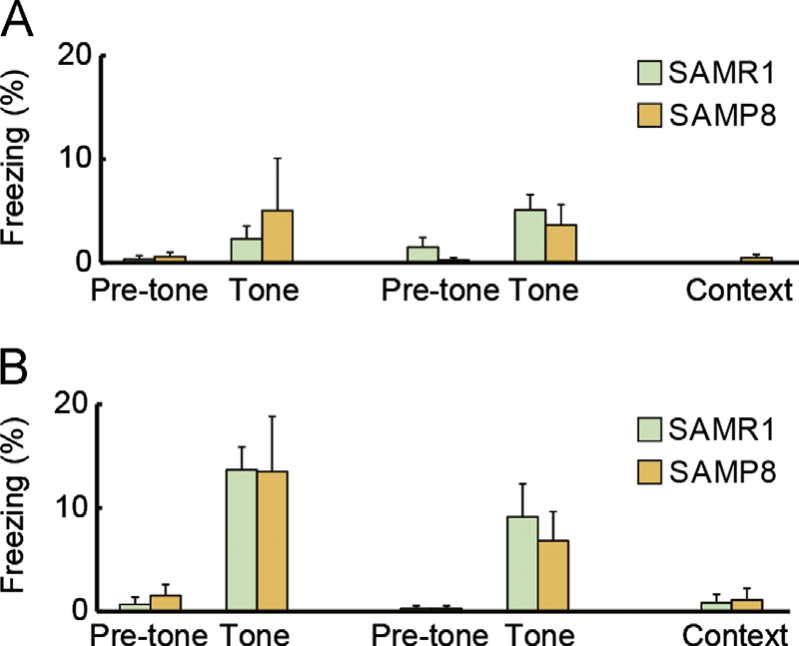
Fear conditioning task. Four-month-old SAMR1 mice (*n*=11) and SAMP8 (*n*=13) were assigned to two groups, and then conditioned fear to tone and context was examined using the conventional conditioning protocol. (A) SAMR1 (*n*=5) and SAMP8 (*n*=7) were conditioned with a CS and 0.12 mA US. (B) SAMR1 (*n*=6) and SAMP8 (*n*=6) were conditioned with CS and 0.30 mA US. For both A and B, conditioned freezing to the tone (1 and 24 h after conditioning) and context (48 h after conditioning) were sequentially measured. Both SAMP8 and SAMR1 displayed relatively little conditioned freezing throughout the entire experiment, even when using different US intensities (Fig. 3; Table 2). Error bars indicate S.E.M.

We examined whether mice had an innate preference for 4 objects in the object preference task (Fig. 1). In the object recognition task (Fig. 2A), we assessed how SAMP8 and SAMR1 habituate to a familiar object (Fig. 2B) in addition to distance traveled (Fig. 2C) throughout the entire experiment. Finally, we assessed whether the typical conditioning protocol for this task [2] induce the conditioned fear in SAMP8 (Fig. 3A,B).

## 2 Experimental design, materials and methods

### 2.1 Object preference task

Four objects (ball, combined disk, cup, and trigonal pyramid; see Fig. 1) were scattered in the arena (50×50 cm arena with transparent wall 50 cm in height), and a single mouse was allowed to explore for 10 min. The goal of this object preference task was to confirm 4 objects to be used in the recognition task for which the mice had no significant innate preference. During this testing period, the mouse׳s object preference was assessed by its contact with the object. Each time the mouse׳s snout or forepaw touched the object, this was counted as a contact [1].

### 2.2 Object recognition task

In the object recognition task (Fig. 2A), we assessed how mice habituated to a familiar object by counting the number of times contact was made with a familiar object, which remained in the same position throughout the entire experiment (Fig. 2B). Further, the distance traveled was analyzed throughout the entire experiment to examine strain differences in terms of locomotor activity (Fig. 2C).

### 2.3 Pavlovian fear conditioning task

Conditioned fear to tone and context was measured according to the procedures described in previous studies [3], [4], [5], [6]. Briefly, mice were placed individually in the conditioning chamber for 60 s before the onset of the conditioned stimulus (CS; 10 kHz, 70 dB tone for 3 s). After conditioning with the CS and the unconditioned stimulus (US; 0.5 s electrical foot shock, 0.12 mA or 0.30 mA), mice were sequentially tested for short-term (1 h) and long-term (24 h) tone-dependent fear memory, followed by a test for context-dependent fear memory (48 h). In the cue-dependent fear memory test, mice were placed in a new chamber and the tone was presented for 60 s. In the context-dependent fear memory test, mice were placed in the original shocking chamber without a foot shock. Throughout the experiments, freezing was used as an index of fear [7].

## 3 Statistical analysis

All data were expressed as means±S.E.M. Statistical differences with regard to strain were assessed by mixed-design two-way analysis of variance (ANOVA) or unpaired *t*-test, as indicated. All statistical analyses were performed using SPSS software (IBM, Tokyo). Statistical significance was set at *p*<0.05. Details of the statistical analyses are provided in Table 1, Table 2.

## Figures and Tables

**Table 1:** Statistical analysis for the object recognition task (mixed-design two-way ANOVA).

Number of contacts with familiar object	
Main effect of strain	*F* (1, 22)=13.08, *p*<0.01
Main effect of trial	*F* (4, 88)=5.04, *p*<0.001
Interaction	*F* (4, 88)=0.92, *p*=0.457

Distance traveled	
Main effect of strain	*F* (1, 22)=0.00, *p*=0.997
Main effect of object	*F* (4, 88)=8.56, *p*<0.001
Interaction	*F* (4, 88)=2.68, *p*<0.05

**Table 2:** Statistical analysis for the Pavlovian fear conditioning task (unpaired *t*-test).

0.12 mA US	

Cue-dependent fear memory test (1 h)	
Pre-tone	*t* (10)=0.47, *p*=0.649
Tone presentation	*t* (10)=0.46, *p*=0.665

Cue-dependent fear memory test (24 h)	
Pre-tone	*t* (10)=1.53, *p*=0.158
Tone presentation	*t* (10)=0.53, *p*=0.610
Context-dependent fear memory test (48 h)	*t* (10)=1.29, *p*=0.226

0.30 mA US	

Cue-dependent fear memory test (1 h)	
Pre-tone	*t* (10)=0.64, *p*=0.540
Tone presentation	*t* (10)=0.03, *p*=0.977

Cue-dependent fear memory test (24 h)	
Pre-tone	*t* (10)=0.00, *p*=1.000
Tone presentation	*t* (10)=0.54, *p*=0.600
Context-dependent fear memory test (48 h)	*t* (10)=0.20, *p*=0.842
